# COVID-19 & informal settlements: is ‘Stay Home’ safe?

**DOI:** 10.14324/111.444/ucloe.000038

**Published:** 2022-07-29

**Authors:** Emily Nix, Jacob Paulose, Monica Lakhanpaul, Pam Factor-Litvak, Priti Parikh, Hector Altamirano-Medina, Yasmin Bou Karim, Logan Manikam, on behalf of the CHIP Consortium

**Affiliations:** 1 UCL Institute for Environmental Design and Engineering, Bartlett School for Energy, Environment and Resources, University College London, London, UK; 2 Population, Policy and Practice, UCL Great Ormond Street Institute of Child Health, London, UK; 3 Whittington Health NHS Trust, London, UK; 4 Department of Epidemiology, Mailman School of Public Health, Columbia University, New York, USA; 5 Engineering for International Development Centre, London, UK; 6 Civil, Environmental and Geomatic Engineering, University College London, London, UK; 7 Aceso Global Health Consultants Ltd, 3 Abbey Terrace, London SE2 9EY, UK

**Keywords:** informal settlements, housing, health, COVID-19

## Abstract

The disproportional burden of coronavirus (COVID-19) and vulnerability to containment measures in informal settlements have been recognised; however, the role of poor housing conditions in propagating these remains neglected. Poor housing conditions makes it difficult to effectively implement social distancing measures. With increased time spent in cramped, dark and uncomfortable indoor environments, water and sanitation outside the home, and no outdoor space, higher exposure to existing health hazards and high levels of stress, with women and children being most vulnerable, are anticipated. Through this commentary paper, we reflect on these interconnections and recommend immediate measures and the long-term need for adequate housing for health and well-being.

## Introduction

Preliminary evidence suggests that those in disadvantaged neighbourhoods, even in high-income countries such as the United States, will experience the largest coronavirus (COVID-19) health burden [1]. Informal settlements are home to some of the most disadvantaged populations globally, where residents face overcrowding, unstable incomes and poor quality housing on vulnerable lands at high risk of climate change impact [2]. One in eight people, or 881 million, live in slums globally; this number is expected to increase to 2 billion with population growth and urban migration over the coming decades, with Asia and Africa expected to see the biggest growth [3]. Conditions in informal settlements are not only likely to contribute to increased COVID-19 transmission risks, due to high contact rates and limited opportunities to practice good hygiene, but infections are likely to be more profound due to co-morbidities, food insecurity and lack of adequate healthcare services [3]. Research estimates that in refugee camps, where housing is similarly limited, large-scale outbreaks of COVID-19 are very likely [4]; however, modelling interventions in these settings suggests that use of face masks and efficient isolation could reduce the incidence of inflections [5]. Although stay-at-home orders are aimed at reducing COVID-19 transmission, such measures are likely to propagate income instability and increase exposure to existing housing-related health hazards in informal settlements.

**Figure 1:**
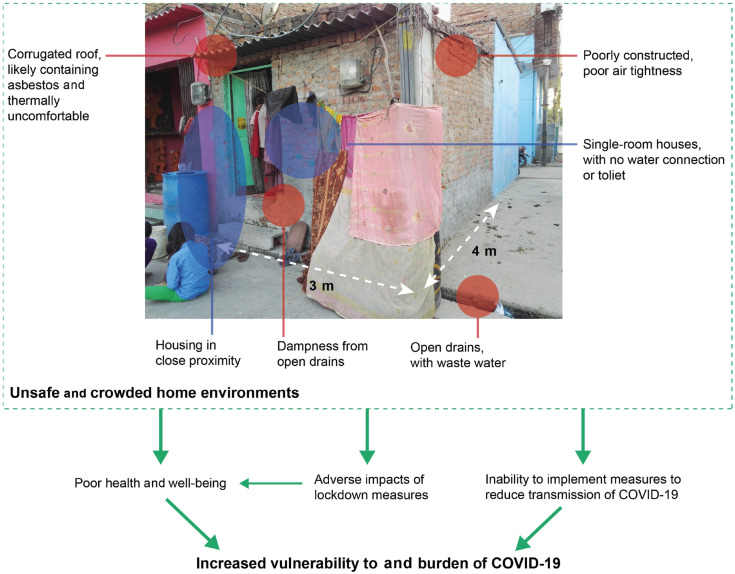
Connections between (non-exhaustive) unsafe and crowded homes environments and COVID-19 vulnerability and burden in informal settlements.

Several published commentaries have discussed how to respond to COVID-19 in informal settlements or similar settings that suffer from poor housing and cramped conditions, such as in refugee camps [6]. For example, Corburn and colleagues [7] provide recommendations for food and income assistance, healthcare services, water, sanitation and waste collection and advise drawing on existing social groups. However, the impact of housing-related deficiencies, specifically, has garnered little attention. Drawing on our field experiences across India’s informal settlements, this commentary examines how housing conditions especially in informal settlements propagate inequalities in 1) the exposure to and 2) the burden of COVID-19 and 3) the unintended impacts of containment measures (Fig. 1). We consider these issues and the longer-term need to consider adequate housing on the global health and development agenda in order to reduce health inequalities and impacts in future pandemics.

### Housing conditions in informal settlements and impacts on health and well-being

Housing in informal settlements tends to be extremely limiting [8]. Plot sizes are very small, typically no more than 15 m^2^, often placed in cramped back-to-back rows with walls and entrances shared with neighbours (Fig. 1). Housing is constructed incrementally, with most housing starting as single-room structures until households can invest in additional floors. Dwellings range from makeshift structures built from bamboo and plastic sheets to substantial structures of bricks walls and concrete roofs, with toilets incorporated where possible. These conditions result in inadequate ventilation and poor lighting levels and insufficient space for occupants, causing dwellings to become polluted and overheat, forcing residents to leave doors open or sit outside – exposing them to mosquitoes and other outdoor risks. Informal settlements generally do not have green space for exercise and well-being, have high levels of environmental pollution or are located on contaminated sites, such as landfills or nearby polluted water sources. Water and sanitation infrastructure are often at a community rather than household level, as is waste collection. Poor urban environment further contributes to the health burden through increased exposure to infections, pollutants and limited ability for healthy lifestyles.

These limiting conditions in informal settlements result in high risks of infection and injury, with children particularly vulnerable, due to malnutrition and recurrent diarrhoea resulting in stunted growth and long-term effects on cognitive development [3]. Previous research in an informal settlement in Delhi revealed how housing: was unable to provide safe indoor temperatures; had poor ventilation; experienced dampness and mould; used hazardous materials and were poorly constructed; experienced overcrowding; had poor lighting and little protection against noise; and suffered from a substantial presence of mosquitoes, pest and food infestation; and facilities for cooking, washing and sanitation were inadequate [8]. Furthermore, this research found that housing had a significant impact on the daily practices of women, as they dealt with the hazards and limiting conditions [8]. The limiting housing and environment conditions will result in health inequalities and increased levels of chronic disease and thus lead to a higher risk of severe outcomes from COVID-19.

## Influence of housing conditions to COVID-19 vulnerability and unintended impacts of containment measures

Housing conditions will significantly limit the ability to implement measures intended to reduce the transmission of COVID-19. With limited indoor space or when people live in only a single room, there is little scope for social isolation within a household. This is an additional burden where multigenerational families stay in the same house, as is typical in India, posing a significant exposure risk to older people who are most vulnerable. There is often less than 2 m between neighbouring entrances, and streets are often less than 2 m wide, making it extremely difficult to maintain distance when leaving the house to collect necessary supplies and to gain access to outdoor air (Fig. 1). Poor air circulation and limited ventilation due to narrow streets are likely to increase the risk of infectious disease, as is found with tuberculosis [9]. Similarly, shared walls with high permeability between dwellings are unlikely to offer protection between households. This situation is further compounded by the frequent need to leave the house to access water sources and use communal toilet spaces; these challenges are discussed in detail in a sister commentary paper [10]. Moreover, with little to no income savings, residents’ ability to stock up on food and other supplies becomes very limited, thereby forcing residents to regularly access public distribution systems or the informal labour market. As a direct result of the home environment, it is very difficult to implement measures to reduce exposure to COVID-19, and thus controlling the spread will be extremely challenging in these settlements.

There are likely to be detrimental effects on health with increased time spent inside in dark, cramped and uncomfortable environments with no or limited private outdoor space. For instance, the current lockdown coincides with India’s peak summer temperatures over 45°C, and with poor housing, temperatures indoors are likely to be higher than the outdoor temperature [8], and if residents are unable to access shade outdoors or cooling appliances the consequences on mortality could be catastrophic. With more occupants remaining indoors, indoor conditions are likely to become hot and stuffy and the ability to move around and complete daily tasks more stressful and result in increased accidents. Furthermore, the risk of contracting other infectious diseases or mosquito-borne diseases (e.g., dengue) could be higher due to increased contact with more people remaining at home. As regards cooking, our previous observations are that children are typically found outside; however, if children remain inside they are likely to have increased exposure to indoor pollution and increased risk of burns and accidents. The small houses provide limited opportunity for productive study or work and the cramped conditions are likely to result in the feeling of suffocation and poor mental health. Women and children are most likely to be affected by these stresses, as a result of an increase in domestic violence – which has been reported globally [11]. The situation is likely to be extremely grave in households with very limited space, as women and children have no respite from their abusers.

## Reflections and recommendations

With widespread social distancing and stay-at-home orders, access to adequate housing and making human settlements safe for health and well-being has never been more important. The COVID-19 pandemic is highlighting the gross inequalities and deficiencies in housing infrastructure. Poor housing conditions surrounding urban form have significant influence not only on vulnerability to COVID-19 but also on potential exposure to COVID-19 and adverse impacts of the lockdown measures.

Improved housing must be part of measures to reduce the burden of infectious diseases and to support the containment of future disease outbreaks. Governments and organisations must start to recognise the role of housing in health inequality and support improved housing for all. Housing quality has too often been missed from the global health agenda, and viewed only through the lens of affordability and access. Current definitions for adequate housing rely on simple characteristics (e.g. whether a house is built from finished materials) which can be not linked to requirements for health [12]. While the recent World Health Organization (WHO) Housing and Health Guidelines [13] are a useful starting place, these must be expanded to include other risks (such as lighting levels, mosquito-borne diseases and pests) and be translated for use in the limiting conditions of informal settlements and be adapted to geographical and social contexts.

In the immediate term, focus needs to be given to those living in the most unsafe dwellings, with remediation measures carried out or alternatives provided. Clean cooking fuel and stoves, mosquito nets and protective equipment should be provided for mitigating both existing risks and increased risks during the COVID-19 pandemic. Cooling appliances, electricity subsidies or access to cool places, such as community centres, should be provided during periods of extreme temperatures and households should not be penalised for leaving their homes. Support should be given to those most vulnerable to COVID-19 and the impacts of lockdown measures, for example, the elderly or women and children at risk of domestic abuse. As outlined elsewhere, action plans should be developed with the local community and agencies with experience in providing support [7]. This approach of local community action, use of mobile clinics and widespread community testing was reported to have successfully curbed COVID-19 within Mumbai’s largest slum [14]. Yet, infection rates were found to be three times higher in Mumbai’s slums compared to other areas [15] and later India faced a huge surge in COVID-19 outbreaks and related deaths [16] – highlighting the long-term need to tackle the poor housing conditions.

Small-scale interventions, such as shading or screens for mosquitoes, can be easily implemented to alleviate some housing deficiencies. Housing design manuals and guidelines should be developed for high-density settings to inform improvements for ventilation, lighting, layouts and other aspects. There needs to be a move away from community-level services in informal settlements and it should be ensured that every household has access to basic amenities at home; COVID-19 highlights the deficiencies in a settlement-level approach. Capacity building programmes are vital to build awareness of health and housing and safe construction practices, appropriate materials and low-cost interventions. Such programmes could form expert community groups that can bridge between the community and government at such times of distress, acting as the frontline workers to better implement and develop effective containment measures.
